# The short-term plasticity of VIP interneurons in motor cortex

**DOI:** 10.3389/fnsyn.2024.1433977

**Published:** 2024-08-29

**Authors:** Amanda R. McFarlan, Isabella Gomez, Christina Y. C. Chou, Adam Alcolado, Rui Ponte Costa, P. Jesper Sjöström

**Affiliations:** ^1^Centre for Research in Neuroscience, Brain Repair, and Integrative Neuroscience Program, Department of Neurology and Neurosurgery, The Research Institute of the McGill University Health Centre, Montreal General Hospital, Montreal, QC, Canada; ^2^Integrated Program in Neuroscience, McGill University, Montreal, QC, Canada; ^3^MTL.AI Inc., Montreal, QC, Canada; ^4^Centre for Neural Circuits and Behaviour, Department of Physiology, Anatomy and Genetics, Medical Sciences Division, University of Oxford, Oxford, United Kingdom

**Keywords:** VIP, inhibitory interneurons, plasticity, short-term plasticity, motor cortex

## Abstract

Short-term plasticity is an important feature in the brain for shaping neural dynamics and for information processing. Short-term plasticity is known to depend on many factors including brain region, cortical layer, and cell type. Here we focus on vasoactive-intestinal peptide (VIP) interneurons (INs). VIP INs play a key disinhibitory role in cortical circuits by inhibiting other IN types, including Martinotti cells (MCs) and basket cells (BCs). Despite this prominent role, short-term plasticity at synapses to and from VIP INs is not well described. In this study, we therefore characterized the short-term plasticity at inputs and outputs of genetically targeted VIP INs in mouse motor cortex. To explore inhibitory to inhibitory (I → I) short-term plasticity at layer 2/3 (L2/3) VIP IN outputs onto L5 MCs and BCs, we relied on a combination of whole-cell recording, 2-photon microscopy, and optogenetics, which revealed that VIP IN→MC/BC synapses were consistently short-term depressing. To explore excitatory (E) → I short-term plasticity at inputs to VIP INs, we used extracellular stimulation. Surprisingly, unlike VIP IN outputs, E → VIP IN synapses exhibited heterogeneous short-term dynamics, which we attributed to the target VIP IN cell rather than the input. Computational modeling furthermore linked the diversity in short-term dynamics at VIP IN inputs to a wide variability in probability of release. Taken together, our findings highlight how short-term plasticity at VIP IN inputs and outputs is specific to synapse type. We propose that the broad diversity in short-term plasticity of VIP IN inputs forms a basis to code for a broad range of contrasting signal dynamics.

## Introduction

Information in the brain is transmitted through synaptic connections between neurons in a dynamic manner. This allows for the activity-dependent modification of synaptic strength between neurons, a concept known as synaptic plasticity. Long lasting changes in synaptic strength are widely believed to underlie learning and information storage in the brain (Bliss and Collingridge, 1993; Malenka and Bear, 2004; Nabavi et al., 2014). But there exist other forms of plasticity, such as short-term facilitation and short-term depression, that occur on a much faster time scale on the order of milliseconds to seconds.

Short-term plasticity is an important feature for processing information and maintaining the balance of excitation and inhibition (E/I) in the brain (Blackman et al., 2013). The initial probability of release dictates the outcome of short-term plasticity at a given synapse. A synapse with a high initial probability of release, for example, will tend to exhibit short-term depression whereas a synapse with a low initial probability of release will tend to exhibit short-term facilitation. Mechanistically, short-term plasticity is primarily governed by changes in the presynaptic terminal, such as synaptic vesicle depletion and the accumulation of calcium in presynaptic terminals (Regehr, 2012). However, postsynaptic factors such as receptor desensitization also contribute to synaptic short-term dynamics (Rozov and Burnashev, 1999; Rozov et al., 2001).

Short-term plasticity varies with a number of different factors. These factors include developmental age (Pouzat and Hestrin, 1997; Cheetham and Fox, 2010), postsynaptic cell type (Wang et al., 2006; Cheetham and Fox, 2010), cortical layer (Reyes and Sakmann, 1999), brain region (Markram et al., 1998; Reyes et al., 1998; Buchanan et al., 2012; Campagnola et al., 2022; Kim et al., 2023), and sensory experience (Finnerty et al., 1999; Cheetham and Fox, 2011; Liu et al., 2012). Despite the various factors that contribute to short-term plasticity outcomes, short-term plasticity tends to be stereotyped for a given synapse type (Blackman et al., 2013). Given the many different cell types that are intermingled in cortical circuits, this synapse specificity leads to a large multiplicity of different forms of short-term dynamics (Campagnola et al., 2022) collectively known as a short-term plasticitome (McFarlan et al., 2023).

Synapse-type specific short-term plasticity rules have been described at synapses between excitatory pyramidal cells (PCs) and inhibitory INs. For example, PC to MC (PC → MC) synapses are short-term facilitating, while PC → BC synapses are short-term depressing (Markram et al., 1998; Reyes et al., 1998; Buchanan et al., 2012). Different short-term plasticity rules have important consequences for information transfer between neurons. For example, facilitating PC → MC synapses are optimally suited for transferring information at high frequencies, whereas depressing PC → BC synapses are optimally suited for transferring information at low frequencies (Blackman et al., 2013).

Though VIP INs play a key disinhibitory role in cortical circuits by inhibiting other IN types, like MCs and BCs, short-term plasticity at synapses to and from VIP INs are not well described. Yet, VIP IN-mediated disinhibition has been shown to boost plasticity and learning in the healthy brain (Fu et al., 2014; Fu et al., 2015; Adler et al., 2019) and has additionally been linked with disease states like epilepsy (Ko et al., 1991; de Lanerolle et al., 1995; Cunha-Reis and Caulino-Rocha, 2020). Indeed, reduced VIP IN inhibitory drive has been shown to have protective effects on seizures in the motor cortex (Khoshkhoo et al., 2017).

We have previously described the synapse-type specific long-term plasticity rules at VIP IN inputs and outputs in the mouse motor cortex (McFarlan et al., 2024), but little is known about the short-term plasticity of VIP IN synapses, especially in this area. Here, we use a combination of whole-cell recording, extracellular stimulation, and optogenetics to describe the short-term dynamics at VIP IN inputs and outputs in the mouse motor cortex.

## Materials and methods

### Animals and ethics statement

This animal study was reviewed and approved by the Montreal General Hospital Facility Animal Care Committee and adhered to the guidelines of the Canadian Council on Animal Care. We crossed homozygous *VIP^tm1(cre)Zjh^*/J mice (JAX strain 010908) with homozygous B6.Cg-Gt(ROSA)26Sor^tm32(CAG-COP4*H134R/EYFP)Hze^/J mice (also known as Ai32, JAX strain 024109) to drive expression of Channelrhodopsin-2 (ChR2) and enhanced yellow fluorescent protein (EYFP) in VIP INs. With this cross, we obtained VIP^Cre/+^; Ai32^flox/+^ mice, henceforth referred to as VIP-ChR2 mice. To carry out our experiments, we used male and female VIP-ChR2 mice from postnatal day (P)20-P45. Mice were anesthetized with isoflurane and were then sacrificed following the loss of the hind-limb withdrawal reflex.

### Acute brain slice electrophysiology

Because the maturity of experimental animals, we optimized slice quality by relying on a sucrose-based cutting solution containing (in mM) 200 sucrose, 2.5 KCl, 1 NH_2_PO_4_, 2.5 CaCl_2_, 1.3 MgCl_2_, 47 D-glucose and 26.2 NaHCO_3_. The solution was bubbled with 95% O_2_/5% CO_2_ for 10 min and cooled on ice to ~4°C. We adjusted osmolality to 338 with glucose, measured using Model 3,300 or Osmo1 osmometers (Advanced Instruments Inc., Norwood, MA, United States).

Following decapitation, the brain was removed and placed in ice-cold sucrose cutting solution. 300-μm-thick coronal acute brain slices were prepared using a Campden Instruments 5,000 mz-2 vibratome (Campden Instruments, Loughborough, United Kingdom) and ceramic blades (Lafayette Instrument, Lafayette, IN, United States). Brain slices were kept at ~33°C in oxygenated artificial cerebrospinal fluid (ACSF), containing (in mM) 125 NaCl, 2.5 KCl, 1 MgCl_2_, 1.25 NaH_2_PO_4_, 2 CaCl_2_, 26 NaHCO_3_ and 25 glucose, bubbled with 95% O_2_/5% CO_2_, for ~10 min and then allowed to cool at room temperature for at least one hour before starting the recordings. ACSF osmolality was adjusted to 338 mOsm with glucose. Throughout experiments, ACSF was heated to 32–34°C with a resistive inline heater (Scientifica Ltd., Uckfield, United Kingdom), with temperature recorded and verified offline. Any recordings with temperatures that fell outside this range were truncated or not used.

An internal solution was prepared containing (in mM) 1 or 5 KCl, 115 K-Gluconate, 10 K-HEPES, 4 Mg-ATP, 0.3 Na-GTP, 10 Na2-Phosphocreatine and 0.1% biocytin. For VIP IN inputs, we used 5 mM KCl in our internal solution. However, to improve signal to noise by increasing the driving force, we used 1 mM KCl for VIP IN outputs. A pH of 7.2 to 7.4 was reached by adding KOH and the target osmolality of 310 mOsm was reached by adjusting with sucrose. 20 μM of Alexa 594 Hydrazide dye (Life Technologies, Eugene, OR, United States) was added to the internal solution to visualize patched cells. We found that postsynaptic responses at VIP IN→BC synapses were occasionally initially depolarizing (*n* = ~10 connections) until the internal solution washed in (~30 min) at which point they became hyperpolarizing. In a subset of experiments with VIP IN inputs, we supplemented the internal solution with 100 μM spermine tetrahydrochloride (Millipore Canada Ltd., Etobicoke, ON, Canada) to explore if polyamine dialysis impacted desensitization of calcium-permeable (CP)-AMPA receptors (Bowie and Mayer, 1995; Donevan and Rogawski, 1995; Lalanne et al., 2016). Patch pipettes had resistances that varied between 4–7 MΩ and were pulled using a P-1000 puller (Sutter Instruments, Novato, CA, United States).

Whole-cell recordings were obtained using BVC-700A amplifiers (Dagan Corporation, Minneapolis, MN, USA). Current clamp recordings were low-pass filtered at 5 kHz and acquired at 40 kHz using PCI-6229 boards (NI, Austin, TX, United States) with custom software (Sjöström et al., 2001; Sjöström et al., 2003) (available at https://github.com/pj-sjostrom/MultiPatch.git) running in Igor Pro 8 or 9 (WaveMetrics Inc., Lake Oswego, OR, United States). We did not account for the liquid junction potential (10 mV), nor did we compensate for series resistance.

We used a LUMPlanFL N 40×/0.80 objective (Olympus, Olympus, Melville, NY, United States) and infrared video Dodt contrast to patch cells on a custom-modified Scientifica SliceScope as previously described (Buchanan et al., 2012). EYFP and Alexa 594 fluorophores were excited using a Chameleon ULTRA II (Coherent, Santa Clara, CA, USA) titanium-sapphire 2-photon (2P) laser tuned to 920 nm or 820 nm, respectively. VIP INs were targeted based on EYFP expression visualized with 2P microscopy at 920 nm. L5 BCs and MCs were targeted based on their small round-shaped soma which were distinctly different from the triangular-shaped soma and prominent apical dendrite that is characteristic of L5 PCs. Cell identity was verified *post hoc* using electrophysiological and morphological properties. The MCs and BCs used in this study were previously included in a dataset published here (McFarlan et al., 2024). As such, the morphological reconstructions of BCs and MCs were not included in this study. Briefly, BCs were characterized based on their fast-spiking and non-accommodating spike pattern, high rheobase, and narrow action potential half width in addition to their densely branching axonal and dendritic arbors. MCs were characterized based on their accommodating spike pattern and lower rheobase along with their stereotypical ascending axon and dangling dendrites (Silberberg and Markram, 2007; Buchanan et al., 2012; Sippy and Yuste, 2013; Tremblay et al., 2016).

### Short-term plasticity experiments

To explore short-term plasticity at VIP IN outputs, L5 BCs and MCs were targeted for whole-cell recording using Dodt contrast in acute slices from P20-P45 VIP-ChR2 mice. L2/3 VIP INs were visualized using 2P microscopy at 920 nm. To activate ChR2-expressing VIP INs in L2/3, we guided a blue laser (1-W 445-nm Blue Laser Diode Module, Item Id: 131542738201, Laserland, eBay.ca) onto the same light path at the 2P beam using a dichroic (FF665-Di02, Semrock Inc., Rochester, NY, United States). The blue laser was controlled with a pair of 6215H 3-mm galvanometric mirrors (Cambridge Technologies, Bedford, MA, United States) and was gated by the MultiPatch software described above. Blue laser pulses had a power of 20 mW and were 2 ms or 5 ms in duration. Due to the lack of single-cell resolution with the blue laser, it is likely that optogenetic stimulation resulted in the activation of more than one presynaptic VIP IN. We previously showed that VIP INs could be reliably driven by blue laser light at frequencies up to 50 Hz (McFarlan et al., 2024). At 30 Hz, a light pulse elicited more than one action potential in ~30% of VIP INs.

For short-term plasticity experiments, we used two protocols: 2 laser pulses delivered at 30 Hz with an inter-stimulus interval of 10 s, repeated between 40 and 75 times or 5 laser pulses delivered at a range of interleaved fixed frequencies (2, 5,10, 20, 30, 40, 50 Hz) with an inter-stimulus interval of 20 s, repeated between 10 and 25 times. To account for temporal summation, we measured IPSP amplitudes as the change in voltage from the IPSP onset to its peak. In a subset of experiments, we accounted for temporal summation more carefully by fitting exponentials, which yielded 7% ± 3% larger PPR values at VIP IN→MC connections (*n* = 8 connections) and 11% ± 8% larger at VIP IN→BC connections (*n* = 8 connections), suggesting a ~ 10% across-the-board underestimation of PPR in our study. This, however, did not affect comparisons across categories, since the PPR underestimation was systematic. Only BCs and MCs that showed inhibitory postsynaptic potentials (IPSPs) >0.2 mV in response to ChR2 activation were used for experiments.

To explore the effect of ChR2 on short-term plasticity outcomes, we targeted L5 MCs and BCs for whole cell recording and activated L2/3 VIP INs with extracellular stimulation. Extracellular stimulation was performed using a Biphasic Stimulation Isolator BSI-950 (Dagan Corporation, Minneapolis, MN, United States) that was manipulated with the MultiPatch software described above. In these experiments, we brought an extracellular stimulating pipette filled with ACSF into the slice in L2/3 of the motor cortex. We used extracellular stimulation pulses that were 100 μs in duration to activate L2/3 VIP INs. A train of 5 stimulation pulses was delivered at 30 Hz with an inter-stimulus interval of 20 s, repeated 20 times. Corresponding IPSPs were recorded in patched L5 BCs and MCs. We blocked excitatory synaptic transmission by bath applying 5 μM of the AMPA receptor blocker NBQX (Hello Bio, Bristol, United Kingdom) throughout experiments.

To explore short-term plasticity at VIP IN inputs, L2/3 VIP INs were targeted for whole-cell recording using 2P microscopy at 920 nm. An extracellular stimulating pipette was brought into the slice ~100–200 μm from the patched VIP IN. We used an input–output curve to measure the excitatory postsynaptic potential (EPSP) response amplitude to incremental increases in extracellular stimulation strength in the patched cell. We used the stimulation strength that yielded EPSPs below the spiking threshold and at least 1 mV in amplitude. We inspected EPSP responses in patched VIP INs to ensure they were due to the activation of VIP IN inputs rather than direct stimulation of the patched VIP IN. In case of direct stimulation, a depolarization response emerged directly from the stimulation artifact, whereas a depolarization onset occurred 1 or 2 ms after the stimulation artifact in the case of a synaptic connection. For short-term plasticity experiments, a train of 5 stimulation pulses was delivered at a range of fixed frequencies in Hz: 2, 5, 10, 20, 30, 40, and 50, with an inter-stimulus interval of 20 s. These frequencies were interleaved to account for any changes in cell properties over the course of the recording and repeated between 10 and 25 times each. In cells that were still healthy following the fixed frequency experiments, we delivered pseudo-random Poisson firing trains consisting of 20 extracellular stimulation pulses at a rate of 5 Hz, repeated 30 times with an inter-stimulus interval of 20 s.

We removed the extracellular stimulation artifact from E → VIP IN synaptic responses. Because many short-term depressing E → VIP IN synapses initially showed paired-pulse facilitation, we relied on the short-term depression (STD) index — calculated the average of EPSP_3 + 4 + 5_ divided by EPSP_1_ — to categorize E → VIP IN synapses as either short-term depressing or facilitating. E → VIP IN synapses with an STD index <1 were categorized as short-term depressing, whereas E → VIP IN synapses with an STD index >1 were categorized as short-term facilitating.

To test whether VIP INs signal via calcium-permeable AMPA receptors, we targeted L2/3 VIP INs for whole-cell recording and activated VIP IN inputs with extracellular stimulation. For these experiments, we supplemented our internal solution with the polyamine spermine. We delivered a train of 5 stimulation pulses at 30 Hz with an inter-stimulus interval of 20 s, repeated 20 times.

To explore whether the heterogeneity in short-term dynamics at E → VIP IN synapses associated with presynaptic inputs or with postsynaptic cells, we targeted L2/3 VIP INs for whole-cell recording and used extracellular stimulation to activate multiple excitatory inputs onto individual VIP INs. At each stimulation site, we delivered a train of 5 stimulation pulses at 30 Hz with an inter-stimulus interval of 20 s, repeated 20 times. We then calculated the PPR for individual E → VIP IN synapses from the average response trace at each stimulation site.

### Identification of motor cortex layers

In acute slices used for electrophysiology experiments, we identified the motor cortex based on the location of the corpus callosum white matter tract. We identified L1 and the white matter based on a relative lack of cell bodies. We used PC morphology to differentiate between L2/3, L5 and, L6. PC somata, for example, are relatively small in L2/3, whereas in L5, PC somata are large and have a thick apical dendrite. PCs in L6 have rounded somata and a thin apical dendrite.

In addition, layer boundaries were informed by *in situ* hybridization (ISH) data from the Allen Institute Mouse Brain Atlas (Lein et al., 2007), as we previously described (McFarlan et al., 2024). Briefly, we selected ISH images with stains for gene markers that were restricted to either L2/3, L4, L5, or L6. Using Fiji/ImageJ (Schindelin et al., 2012), we measured the intensity profile of a region of interest that spanned from pial surface to white matter in the motor cortex and then overlayed the intensity profiles for each gene marker. We used the point of intersection between pixel intensity profiles to define layer boundaries.

### Biocytin histology and morphological reconstructions

In acute 300-μm-thick coronal slices from VIP-ChR2 mice, patched L2/3 VIP INs used in short-term plasticity experiments were saved for neuronal reconstructions. Once the experiment was completed, the patch pipette was removed slowly while lightly applying positive pressure. Sections were then incubated in 4% paraformaldehyde overnight and were stored in a 0.01 M phosphate buffer solution for up to 3 weeks before staining.

Sections were placed in 0.01 M Tris-buffered saline (TBS) solution with 0.3% Triton-X for four ten-minute washes. Then, sections underwent a one-hour wash in 0.01 M TBS with 0.3% Triton-X and 10% normal donkey serum (NDS; 017–000-121 Jackson ImmunoResearch, West Grove, PA, United States) followed by an overnight incubation at 4°C in 0.01 M TBS with 0.3% Triton-X and 1% NDS, supplemented with 1:200 Alexa Fluor 647- or Alexa fluor 488-conjugated Streptavidin (ThermoFisher Scientific, Waltham, MA, United States). The next day, tissue underwent four ten-minute washes in 0.01 M TBS. Next, sections were mounted using coverslips with a 40 μL bolus of ProLong Gold Antifade Mountant (ThermoFisher Scientific). We acquired 3D image stacks using a Zeiss LSM780 confocal laser scanning microscope and ZEN software (Zeiss). These 3D stacks were then used for morphological reconstructions.

3D confocal image stacks were contrast adjusted and converted to 16 bits in Fiji (Schindelin et al., 2012) and were then imported into Neuromantic V1.7.5 (Myatt et al., 2012) to be manually traced. Morphometry was performed in Igor Pro 9 (Wavemetrics) using the qMorph in-house custom software as previously described (Buchanan et al., 2012; Zhou et al., 2021) (available at https://github.com/pj-sjostrom/qMorph).

### Phenomenological modeling of short-term plasticity data

We used our fixed-frequency short-term plasticity data at excitatory inputs onto VIP INs to tune a 2-parameter Tsodyks-Markram (TM) vesicle depletion short-term depression model, as well as a 3-parameter and 4-parameter TM model extended with short-term facilitation (Markram et al., 1998; Tsodyks et al., 1998). Tuning was done with Bayesian inference as previously described (Costa et al., 2013) (https://github.com/neuralml/STPinference). Unless stated otherwise, the code was implemented in Matlab (MATLAB version: 9.14.0 (R2022a), The MathWorks Inc., Natick, Massachusetts, United States).

The phenomenological model was defined by the following ordinary differential equations:


(1) $$\frac{dR\left(t\right)}{dt}=\frac{1-R\left(t\right)}{D}-u\left(t\right)\delta \left(t-t_{AP}\right)$$

(2) $$\frac{du\left(t\right)}{dt}=\frac{U-u\left(t\right)}{F}+f\left[1-u\left(t\right)\right]\delta \left(t-t_{AP}\right)$$

The vesicle depletion process is modeled in Equation 1 where the number of vesicles $R\left(t\right)$ is decreased with $u\left(t\right)R\left(t\right)$ after release due to a presynaptic spike at time $t_{AP}$ , modeled by a Dirac delta, $\delta \left(t\right)$ . $R\left(t\right)$ recovers to 1 between spikes with a depression time constant D . Equation 2 models the dynamics of the release probability $u\left(t\right)$ which increases with $f\left[1-u\left(t\right)\right]$ after every presynaptic spike, decaying back to baseline release probability U with a facilitation time constant F .

It is possible to obtain depressing, combined facilitating-depressing, as well as facilitating synaptic dynamics by varying the four parameters $\theta =\left\{D,F,U,f\right\}$ . We tested three variants of the model. First, the full 4-parameter model. Then a 3-parameter model in which we set f=U , which is the TM model with facilitation. Finally, a 2-parameter depression-only model with only Equation 1, in which we set $u\left(t\right)=U$ .

We sped up the numerical implementation by integrating the Equations 1 and 2 between spikes n and n+1 , a time $\Delta t_{n}$ apart, which yielded


(3) $$R_{n+1}=1-\left[1-R_{n}\left(1-u_{n}\right)\right]\exp\left(-\frac{\Delta t_{n}}{D}\right)$$

(4) $$u_{n+1}=U+\left[u_{n}+f\left(1-u_{n}\right)\right]\exp\left(-\frac{\Delta t_{n}}{F}\right)$$

We assumed that the synapse was not recently activated at time zero, and therefore set $R_{0}=1$ and $u_{0}=U$ .

The postsynaptic potential $PSP_{n}$ is given by:


(5) $$PSP_{n}=AR_{n}u_{n}$$

where A is an amplitude factor that includes the number of release sites, the properties and number of postsynaptic receptors, and cable filtering.

### Model validation

We validated our models tuned to fixed-frequency EPSP trains by testing their predictions on pseudo-random Poisson EPSP trains, as previously described (Varela et al., 1997). Electrophysiological recordings with Poisson trains that exhibited changes in membrane potential >8 mV, input resistance >30%, or EPSP amplitude >30% relative to fixed-frequency experiments were excluded from the model validation.

### Model selection

We used several metrics to assess the goodness of fit of the 2-parameter, 3-parameter, and 4-parameter models. The first metric was the Akaike Information Criterion (AIC), which is a measure of the goodness of fit for a given statistical model. It is defined as $AIC=2k+n*\ln\left(SSe/n\right)$ , where *k* is the number of estimable parameters in the model, SSe is the sum of squared errors, and n is the number of observations. AIC values were calculated in excel. We used relative ranking of the Akaike weights to find the least complex model that best describes the data.

The second metric was the *R*^2^ goodness of fit test, which measures how well the variation in the dependent variable is explained by a linear regression model. Higher *R*^2^ values imply that a larger proportion of variance can be explained by the model and therefore indicate a better fit. *R*^2^ was calculated in Matlab.

The third metric was the root mean squared (RMS) error, which is a measure of the average difference between predicted and measured values. Smaller RMS error thus indicates a better fit. RMS error values were calculated in Matlab.

The final metric was the Kolmogorov–Smirnov (KS) test, which is a non-parametric test that can be used to determine how well a sample data distribution matches a theoretical distribution. A small KS test *p*-value indicates that the fit between sample and theory is poor. The KS test was done in JMP (JMP Pro Version 17, SAS Institute Inc., Cary, NC, United States).

### Statistics

Unless otherwise stated, results are reported as the mea*n* ± standard error of the mean (SEM). Boxplots indicate median and quartiles by Tukey’s method, with whiskers drawn to data point extremes. Significance levels are denoted using asterisks (* *p* < 0.05, ** *p* < 0.01, *** *p* < 0.001). Pairwise comparisons were carried out using a two-tailed Student’s t-test for equal means. If the equality of variances *F* test gave p < 0.05, we used the unequal variances t-test. We took the logarithm of PPR and STD index prior to statistical tests that required normality. However, for the rheobase current, which was discretized, we employed the non-parametric Wilcoxon-Mann–Whitney test. Statistical tests were performed in Igor Pro 9 (Wavemetrics) and JMP, unless otherwise stated.

We used linear mixed models (LMMs) running in RStudio 2023.06.1.524 (Posit Software, Boston, MA) (PositTeam, 2023) to explore the dependency of log PPR on synapse type, frequency, experimental condition, and age. We employed an LMM because individual data points (i.e., PPRs) were obtained from the same postsynaptic cell or the same animal and were therefore not independent. As such, we included individual postsynaptic cells nested in individual animals as a random factor. We fitted an LMM to log PPR using the lme() function from the nlme package (Pinheiro and Bates, 2000). With the lme() function, the restricted maximum likelihood method is used to estimate LMM statistics. To test the significance of fixed effects (synapse type, frequency, experimental condition, and age), we carried out F tests using the fitted model as the argument in the anova() function from the stats package. If fixed effects were significant, we carried out pairwise comparisons using the emmeans() function from the emmeans package, which uses the Tukey method to adjust *p*-values for multiple comparisons.

To assess whether VIP → BC and VIP → MC synapses exhibited STD, we fitted single-mean LMMs to log PPR measured via ChR2 stimulation and extracellular stimulation at VIP → BC and VIP → MC synapses. We included postsynaptic cells nested in individual animals as a random factor. LMM statistics revealed that the estimated average logged PPR was significantly different from 0.

To determine whether the release probability parameter *U* was correlated with STP outcomes, we computed the Pearson correlation coefficient between log STD index and *U*. We used the average log STD index across all tested frequencies for each cell.

## Results

### Short-term plasticity at VIP IN outputs

#### VIP IN outputs had differing kinetics

**Figure 1:**
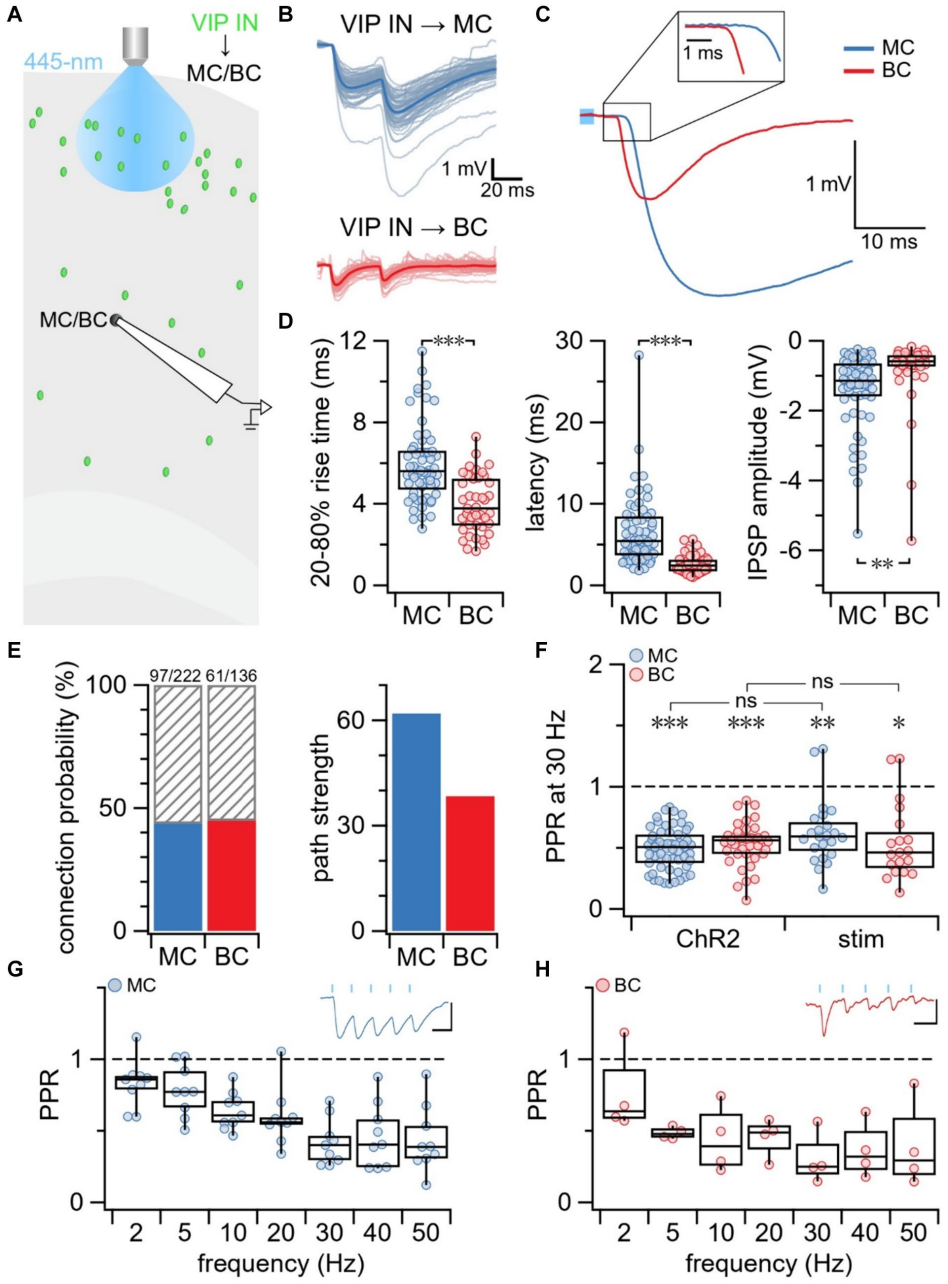
Short-term depression at VIP IN outputs onto MCs and BCs. **(A)** Schematic illustrating the experimental paradigm. MCs and BCs were targeted for whole-cell recording in L5 of the mouse motor cortex. L2/3 VIP INs were activated with blue laser light which resulted in IPSPs in connected MCs and BCs. **(B)** Sample traces illustrating recorded IPSPs in a MC and BC following stimulation delivered with blue laser light. Average traces are in blue (MC) and red (BC) while individual responses are in light-blue (MC) and pink (BC). **(C)** Overlay of average IPSP traces (from B) aligned with respect to onset of blue laser light pulse (light blue square). The initial phase of the IPSP is shown at a higher resolution as an inset. **(D)** VIP IN→MC and VIP IN→BC connections differed in kinetics. Mea*n* ± SEM was calculated for VIP IN→MC (*n* = 60 connections, *N* = 53 animals) and VIP IN→BC (*n* = 41 connections, *N* = 35 animals) groups. (Left) Compared to VIP IN→MC synapses, VIP IN→BC had faster 20–80% rise time (VIP IN→MC: 6.0 ms ± 0.2 ms vs. VIP IN→BC: 4.0 ms ± 0.2 ms, unequal variances *t*-test *p* < 0.001) and (middle) shorter latency (VIP IN→MC: 6.6 ms ± 0.6 ms vs. VIP IN→BC: 2.6 ms ± 0.2 ms, unequal variances *t*-test *p* < 0.001). (Right) IPSP amplitude was larger at VIP IN→MC compared to VIP IN→BC synapses (VIP IN→MC: −1.4 mV ± 0.1 mV vs. VIP IN→BC: −0.86 mV ± 0.2 mV, *t*-test *p* < 0.01). Rise time, latency, and amplitude were analyzed based on averages from individual connections. **(E)** Connection probability (left) was similar for VIP IN→MC connections (97/222, 44%) and VIP IN→BC connections (61/136, 45%). Path strength (right), however, was almost twice as strong for VIP IN→MC compared to VIP IN→BC connections (VIP IN→MC: 62 vs. VIP IN→BC: 38). **(F)** PPR revealed that VIP IN→MC synapses and VIP IN→BC synapses were short-term depressing when activating L2/3 VIP INs with blue laser light (ChR2: VIP IN→MC: 0.49 ± 0.02, *n* = 58 connections, *N* = 51 animals; VIP IN→BC: 0.53 ± 0.03, *n* = 40 connections, *N* = 34 animals) and with extracellular stimulation (stim: VIP IN→MC: 0.54 ± 0.07, *n* = 19 connections, *N* = 3 cells; VIP IN→BC: 0.62 ± 0.06, *n* = 23 connections, *N* = 5 cells). LMM statistics revealed that PPR did not differ between methods for VIP IN→MC synapses (*p* = 0.69) or for VIP IN→BC synapses (*p* = 0.31). **(G)** PPR revealed that VIP IN→MC synapses exhibited short-term depression at all tested frequencies (PPR at 2 Hz: 0.82 ± 0.06, 5 Hz: 0.78 ± 0.06, 10 Hz: 0.64 ± 0.04, 20 Hz: 0.59 ± 0.07, 30 Hz: 0.42 ± 0.05, 40 Hz: 0.46 ± 0.07, 50 Hz: 0.44 ± 0.08). Top right inset: Sample trace of VIP IN→MC IPSPs due to 5 blue light pulses (blue bars) delivered at 20 Hz (*n* = 9 cells, *N* = 8 animals). X-axis scale bar: 50 ms; y-axis scale bar: 1 mV. **(H)** PPR revealed that VIP IN→BC synapses exhibited short-term depression at all tested frequencies (PPR at 2 Hz: 0.76 ± 0.1, 5 Hz: 0.48 ± 0.02, 10 Hz: 0.44 ± 0.1, 20 Hz: 0.45 ± 0.07, 30 Hz: 0.30 ± 0.09, 40 Hz: 0.36 ± 0.1, 50 Hz: 0.39 ± 0.2). Top right inset: Sample trace of VIP IN→BC IPSPs due to 5 blue light pulses (blue bars) delivered at 20 Hz (*n* = 4 cells, *N* = 4 animals). X-axis scale bar: 50 ms; y-axis scale bar: 0.2 mV.

To study short-term plasticity at VIP IN outputs, we used a combination of optogenetics and electrophysiology. We previously showed that the expression of ChR2 in our VIP-ChR2 mice was highly specific to VIP INs (McFarlan et al., 2024). Since VIP INs chiefly form synapses with other IN types (Pfeffer et al., 2013; Kepecs and Fishell, 2014; Tremblay et al., 2016), namely MCs and BCs, we investigated short-term plasticity at these two synapse types. In the motor cortex of our VIP-ChR2 mice, we targeted postsynaptic L5 MCs and BCs for whole-cell recording and selectively activated L2/3 VIP INs with blue laser light (Figure 1A). MCs and BCs were identified based on electrophysiology and morphometry (see Methods). Compared to VIP IN→MC synapses, IPSPs at VIP IN→BC synapses had a shorter rise-time (unequal variances t-test *p* < 0.001) and latency (unequal variances *t*-test *p* < 0.001; Figures 1B–D). On average, the IPSP amplitude was larger at VIP IN→MC connections compared to VIP IN→BC connections (*t*-test *p* < 0.01, Figures 1B–D), even when accounting for resting membrane potential (normalized IPSP amplitude at VIP IN→MC: 2.4 ± 0.2 vs. VIP IN→BC: 1.5 ± 0.3, *t*-test *p* < 0.05). VIP IN→MC and VIP IN→BC synapses had similar connectivity rates (VIP IN→MC: 44% vs. VIP IN→BC: 45%, Figure 1E). However, the path strength, which is calculated as the product of IPSP amplitude and connection probability, was stronger at VIP IN→MC synapses compared to VIP IN→BC synapses (Figure 1E).

#### VIP IN outputs were short-term depressing

To explore short-term plasticity at VIP IN outputs, we measured the PPR. We found that both VIP IN→MC and VIP IN→BC synapses were short-term depressing at 30 Hz (Figure 1F). Prior studies have suggested that using 1-photon optogenetics to activate ChR2 —which is known to flux many types of cations including calcium (Yang et al., 2019)— may artificially increase the probability of release and skew short-term dynamics toward depression (Zhang and Oertner, 2007; Cruikshank et al., 2010; Jackman et al., 2014). Indeed, studies using ChR2 to study short-term dynamics have reported depression at VIP IN→MC and VIP IN→BC synapses (Pi et al., 2013), whereas studies using paired recordings have reported short-term depression at VIP IN→BC synapses and short-term facilitation at VIP IN→MC synapses (Walker et al., 2016; Campagnola et al., 2022).

#### ChR2 did not affect short-term plasticity outcomes

To address this possible caveat with using ChR2, we patched L5 MCs and BCs and activated L2/3 VIP INs with extracellular stimulation while blocking excitatory neurotransmission with bath application of the AMPA receptor blocker NBQX (Supplementary Figure S1A). Similar to ChR2-evoked IPSPs at VIP IN→MC and VIP IN→BC synapses, we found that IPSP amplitude was larger at VIP IN→MC connections compared to VIP IN→BC connections (*t*-test *p* < 0.01, Supplementary Figures S1B,C). Likewise, IPSPs at VIP IN→BC synapses had a shorter rise-time (*t*-test, *p* < 0.001) and latency (*t*-test, *p* < 0.001) compared to IPSPs at VIP IN→MC synapses (Supplementary Figures S1D,E).

LMM statistics revealed that extracellular activation of VIP INs resulted in paired-pulse depression at both VIP IN→MC and VIP IN→BC synapses that was indistinguishable from short-term depression observed using ChR2 (VIP IN→MC: *p* = 0.69; VIP IN→BC: *p* = 0.31; Figure 1F). Thus, we concluded that the presence of ChR2 at the synapse did not have an effect on short-term plasticity outcomes at VIP IN outputs.

#### VIP IN outputs were consistently depressing at frequencies up to 50 Hz

Prior literature has shown that short-term plasticity outcomes at VIP IN→MC synapses can be influenced by presynaptic stimulation frequency (Walker et al., 2016). We therefore explored if short-term plasticity at VIP IN outputs depended on frequency. We optogenetically activated L2/3 VIP INs with 5 laser pulses delivered at frequencies between 2 and 50 Hz and recorded IPSPs in patched L5 MCs and BCs. We found that the PPR at VIP IN→MC (Figure 1G) and VIP IN→BC (Figure 1H) synapses was short-term depressing at all tested frequencies.

### Short-term plasticity at VIP IN inputs

#### Excitatory inputs to VIP INs had heterogeneous short-term dynamics

**Figure 2:**
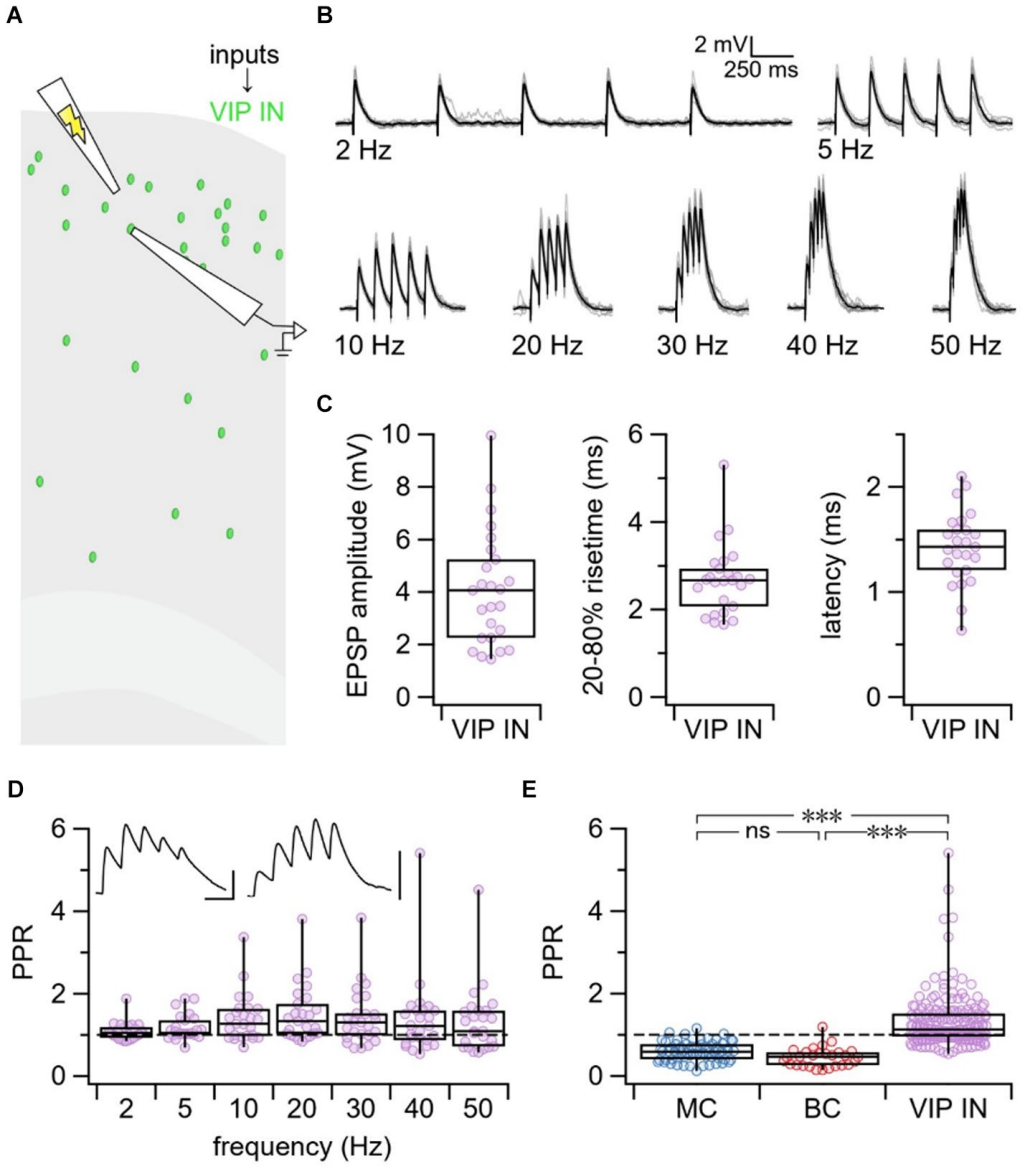
VIP IN inputs exhibited heterogeneous short-term dynamics. **(A)** Schematic illustrating the experimental paradigm. VIP INs were targeted for whole-cell recording in L2/3 of the motor cortex. EPSPs from local excitatory inputs onto VIP INs were generated using extracellular stimulation and were recorded in the patched VIP INs. **(B)** Example traces from a patched VIP IN illustrating EPSPs in response to 5 pulses of extracellular stimulation delivered at 2, 5, 10, 20, 30, 40, and 50 Hz. Gray traces represent individual responses and black trace represents the average response. **(C)** On average, E → VIP IN synapses had an EPSP amplitude of 4.1 mV ± 0.4 mV, a 20–80% rise-time of 2.7 ms ± 0.2 ms, and a latency of 1.4 ms ± 0.1 ms (*n* = 25 cells, *N* = 18 animals). **(D)** PPR at all tested frequencies revealed that E → VIP IN synapses had diverse short-term dynamics (PPR at 2 Hz: 1.1 ± 0.04, 5 Hz: 1.2 ± 0.1, 10 Hz: 1.4 ± 0.1, 20 Hz: 1.5 ± 0.1, 30 Hz: 1.4 ± 0.1, 40 Hz: 1.4 ± 0.2, 50 Hz: 1.3 ± 0.2). Top left inset: Sample traces of short-term depressing (left) and short-term facilitating (right) E → VIP IN connections. Scale bars: 50 ms, 3 mV. **(E)** LMM statistics revealed that PPR at E → VIP IN synapses was different compared to PPR at VIP IN→MC and VIP IN→BC synapses (*p* < 0.001). In this figure panel, PPR data was pooled across all tested frequencies for each synapse type.

Next, we targeted L2/3 VIP INs for whole-cell recording and used extracellular stimulation to activate local excitatory inputs onto VIP INs. We delivered 5 pulses at varying frequencies from 2 to 50 Hz to explore short-term plasticity at E → VIP IN synapses (Figures 2A,B). The EPSP amplitude, rise-time, and latency were calculated based on the average response trace for each individual connection (Figure 2C). Although the PPR revealed more facilitation (Figure 2D), there was a surprising diversity in short-term dynamics at E → VIP IN synapses compared to VIP IN outputs (Figure 2E). Using an LMM, we revealed an interaction effect between synapse type and frequency (p < 0.001). PPR was different at VIP IN inputs compared to outputs for all tested frequencies between 5 Hz and 50 Hz, but not for 2 Hz.

#### VIP INs with short-term facilitating and depressing inputs were indistinguishable

To explore why E → VIP IN synapses were heterogeneous in their short-term dynamics, we first hypothesized that there could be differences in basic properties of the patched VIP INs. However, we found that VIP INs with depressing vs. facilitating inputs were indistinguishable with respect to morphology, spike pattern, and electrophysiological properties (Supplementary Figure S2; Supplementary Table S1). We next thought this diversity may be attributed to age, however, there was no correlation between PPR and age (*p* > 0.05, Supplementary Figure S3).

#### E → VIP IN synapses did not signal via calcium-permeable AMPA receptors

Next, we speculated that accidental and uncontrolled polyamine dialysis during experiments might cause variable desensitization of CP-AMPA receptors at VIP IN inputs, which could explain the heterogeneous short-term dynamics we have observed (Rozov and Burnashev, 1999). To test whether E → VIP IN connections signal via CP-AMPA receptors, we supplemented our internal patch solution with the polyamine spermine and stimulated presynaptic VIP IN inputs with 5 pulses delivered at 30 Hz (Supplementary Figure S4A). We found that supplementing with spermine had no effect on short-term dynamics (Wilcoxon-Mann–Whitney test *p* = 0.33, Supplementary Figure S4B), arguing against this possibility.

#### Short-term plasticity heterogeneity at E → VIP IN synapses linked to target VIP IN cell

**Figure 3:**
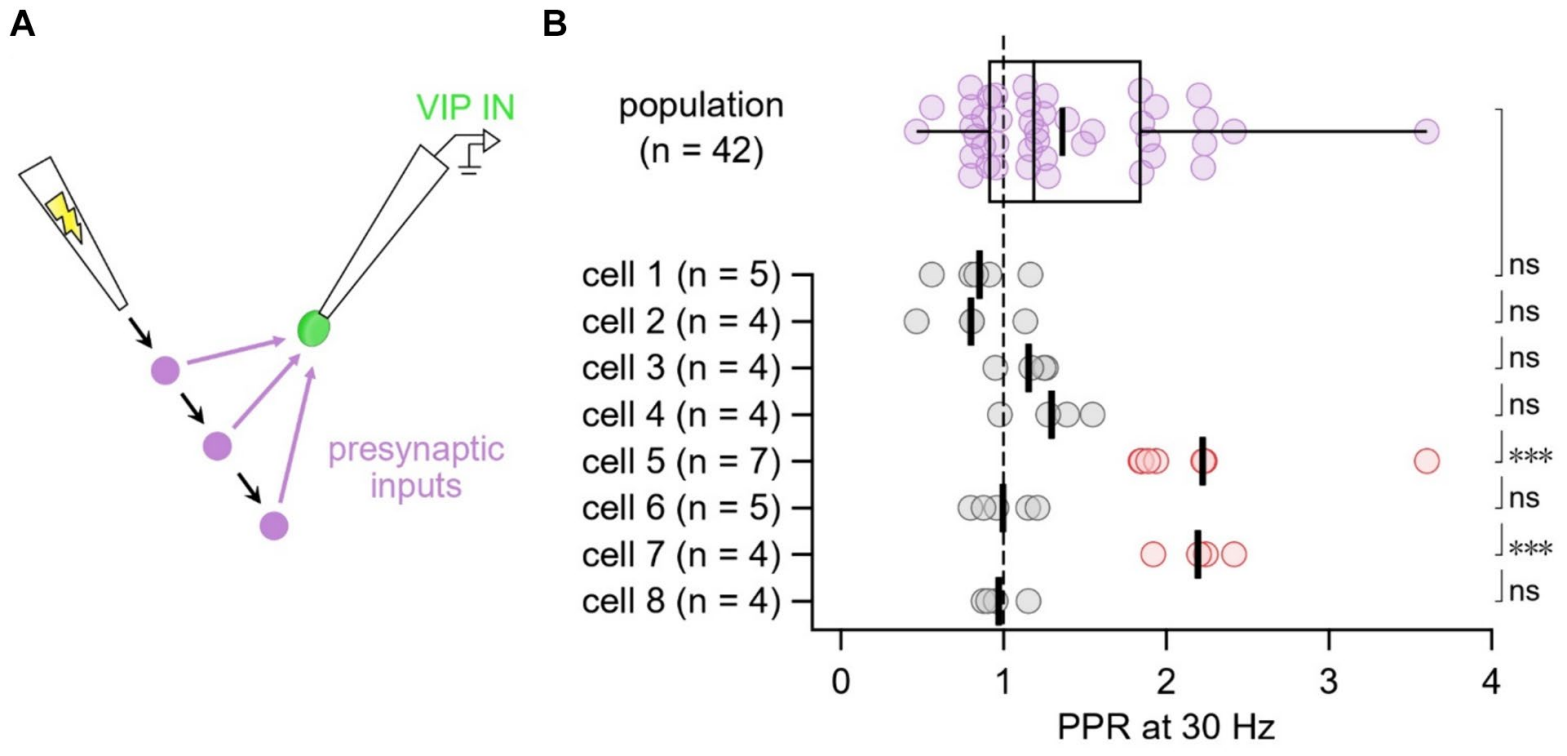
E → VIP IN short-term dynamics linked with target cell rather than with synaptic input **(A)** In this experimental paradigm, we targeted VIP INs for whole-cell recording and moved the extracellular stimulation pipette to several different positions in the slice to activate multiple presynaptic inputs onto individual VIP INs. **(B)** Compared to the population average, cells 5 and 7 (red) short-term facilitated (one-tailed t-test of log PPR), an outcome that data shuffling demonstrated was unlikely (18 such outcomes across 100,000 shuffled trials, implying *p* < 0.001). In conclusion, short-term dynamics associated with the postsynaptic cell rather than with the synaptic inputs. Thick vertical lines: mean.

We then explored whether the heterogeneity in short-term dynamics at E → VIP IN synapses associated with presynaptic inputs or with postsynaptic cells. To test this, we targeted L2/3 VIP INs for whole-cell recording and used extracellular stimulation to activate multiple excitatory inputs onto individual VIP INs (Figure 3A). We found that two cells were strongly short-term facilitating compared to the population average, an outcome that data shuffling furthermore suggested was unlikely (Figure 3B). Thus, we associated PPR heterogeneity with the target VIP IN cell rather than with the input.

### Computational modeling of short-term plasticity at VIP IN inputs

#### VIP IN inputs showed a wide variability in probability of release

**Figure 4:**
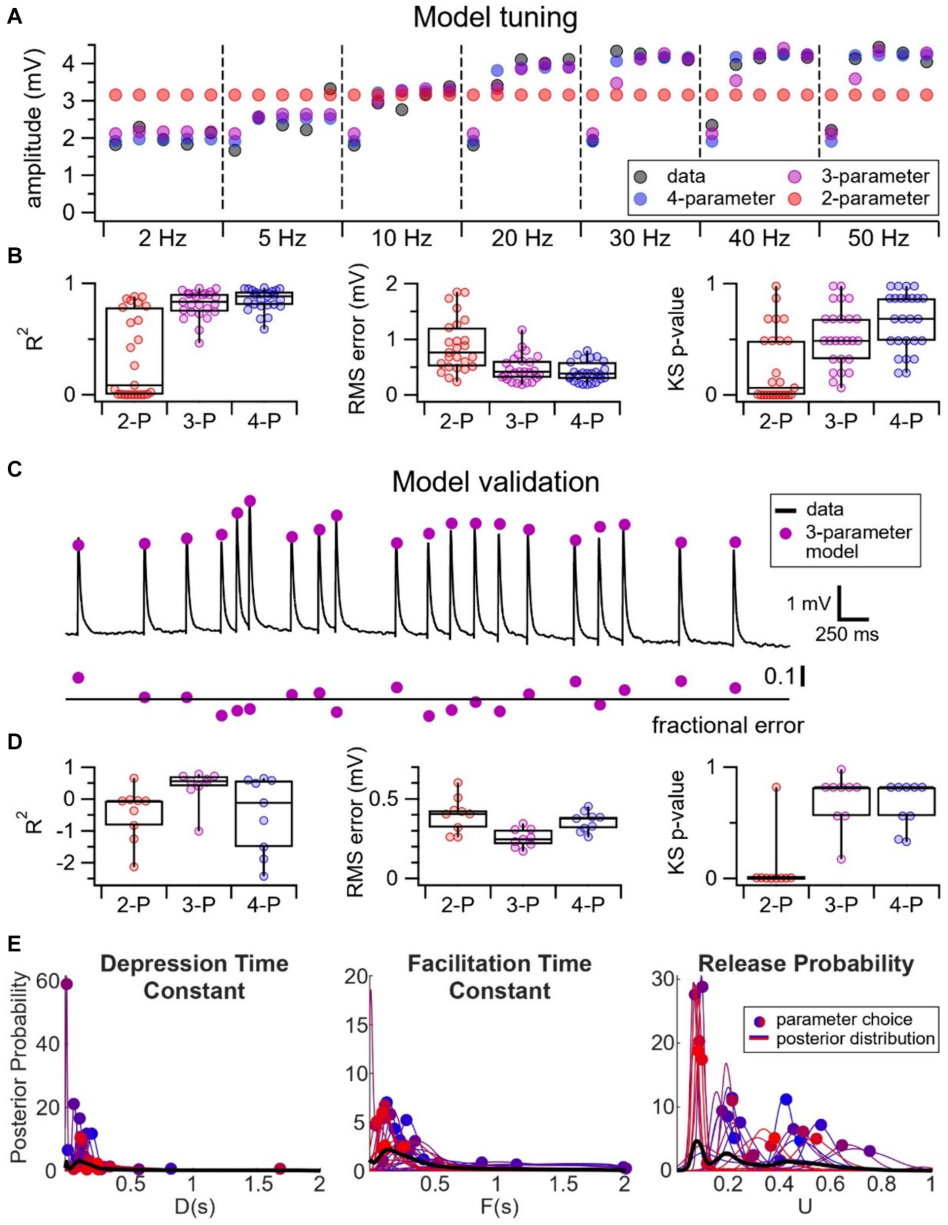
Wide variability in probability of release at E → VIP IN synapses. **(A)** Sample experiment illustrating the tuning of a 2-parameter (red) vs. 3-parameter (purple) vs. 4-parameter (blue) model to our fixed frequency short-term plasticity data (black). Due to the presence of short-term facilitation, the 2-parameter model — which only has short-term depression — performs poorly. **(B)** *R*^2^ goodness of fit (2-P: 0.34 ± 0.08; 3-P: 0.81 ± 0.02; 4-P: 0.85 ± 0.02), RMS error (2-P: 0.89 mV ± 0.1 mV; 3-P: 0.47 mV ± 0.05 mV; 4-P: 0.41 mV ± 0.04 mV), and KS test (*p*-values: 2-P: 0.25 ± 0.06; 3-P: 0.52 ± 0.05; 4-P: 0.67 ± 0.05) revealed that the 4-parameter and 3-parameter models fit the data better than the 2-parameter model (*n* = 25 cells, *N* = 18 animals). **(C)** We validated our models using pseudo-random Poisson spike trains. Here, black traces represent a sample Poisson spike train and the purple circles represent the EPSP predictions using the 3-parameter model. The model fit is indicated by the fractional error, calculated as the difference between observed and predicted EPSPs, divided by observed EPSPs. **(D)** *R*^2^ goodness of fit (2-P: −0.46 ± 0.3; 3-P: 0.40 ± 0. 2; 4-P: −0.48 ± 0.4;), RMS error (2-P: 0.40 mV ± 0.04 mV; 3-P: 0.25 mV ± 0.02 mV; 4-P: 0.36 mV ± 0.02 mV), and KS test (*p*-values: 2-P: 0.093 ± 0.09; 3-P: 0.71 ± 0.08; 4-P: 0.65 ± 0.07) revealed that the 3-parameter model best predicted the EPSPs for the Poisson spike trains (*n* = 9 Poisson, *N* = 4 cells). **(E)** The 3-parameter TM model revealed that the depression time constant, *D*, and the facilitation time constant, *F*, distributed tightly, whereas there was a relatively broad range of values of the release probability, *U*.

To further elucidate which components of presynaptic release machinery — e.g., probability of release, depression recovery rate, or facilitating recovery rate — contributed to the heterogeneity in short-term dynamics at VIP IN inputs, we relied on computational modeling. We tuned a 2-parameter TM vesicle depletion short-term depression model, as well as a 3-parameter and 4-parameter TM model extended with short-term facilitation to our fixed frequency short-term plasticity data at E → VIP IN synapses (Figure 4A). Tuning was done with Bayesian inference (Costa et al., 2013; Ola et al., 2019). Unsurprisingly, the 4-parameter and 3-parameter models fit our data better than the 2-parameter model, as indicated by AIC, *R*^2^ goodness of fit, RMS error, and KS test (Figure 4B; see Methods). We surmised that the better fit could in part be attributed to the 3-parameter and 4-parameter models accounting for the heterogeneity of short-term facilitation at E → VIP IN synapses.

We then validated our model by testing its predictions using pseudo-random Poisson trains (Figure 4C). Here, the 3-parameter model performed the best, as indicated by AIC, *R*^2^ goodness of fit, RMS error, and KS test, perhaps because the 4-parameter model overfit the fixed-frequency training data. We concluded that the 3-parameter model was the least complex model that best described our data (Figure 4D).

To determine if a specific component of the release machinery underpinned the diversity in short-term plasticity at E → VIP IN synapses, we looked at the distribution of the probability of release as well as the depression and facilitation recovery rates of the 3-parameter TM model. Here, we opted to use the 3-parameter TM model because it described our dataset best (Figure 4D). The 3-parameter TM model revealed that the depression and facilitation recovery rates distributed tightly, whereas there was a relatively wide distribution of the parameter *U* that described release probability (Figure 4E). Pearson’s correlation additionally revealed that the probability of release correlated with STP outcomes (*r* = −0.64, *p* < 0.001). These findings thus suggest that the probability of release was a key determinant of the heterogeneity of E → VIP IN short-term dynamics.

## Discussion

**Figure 5:**
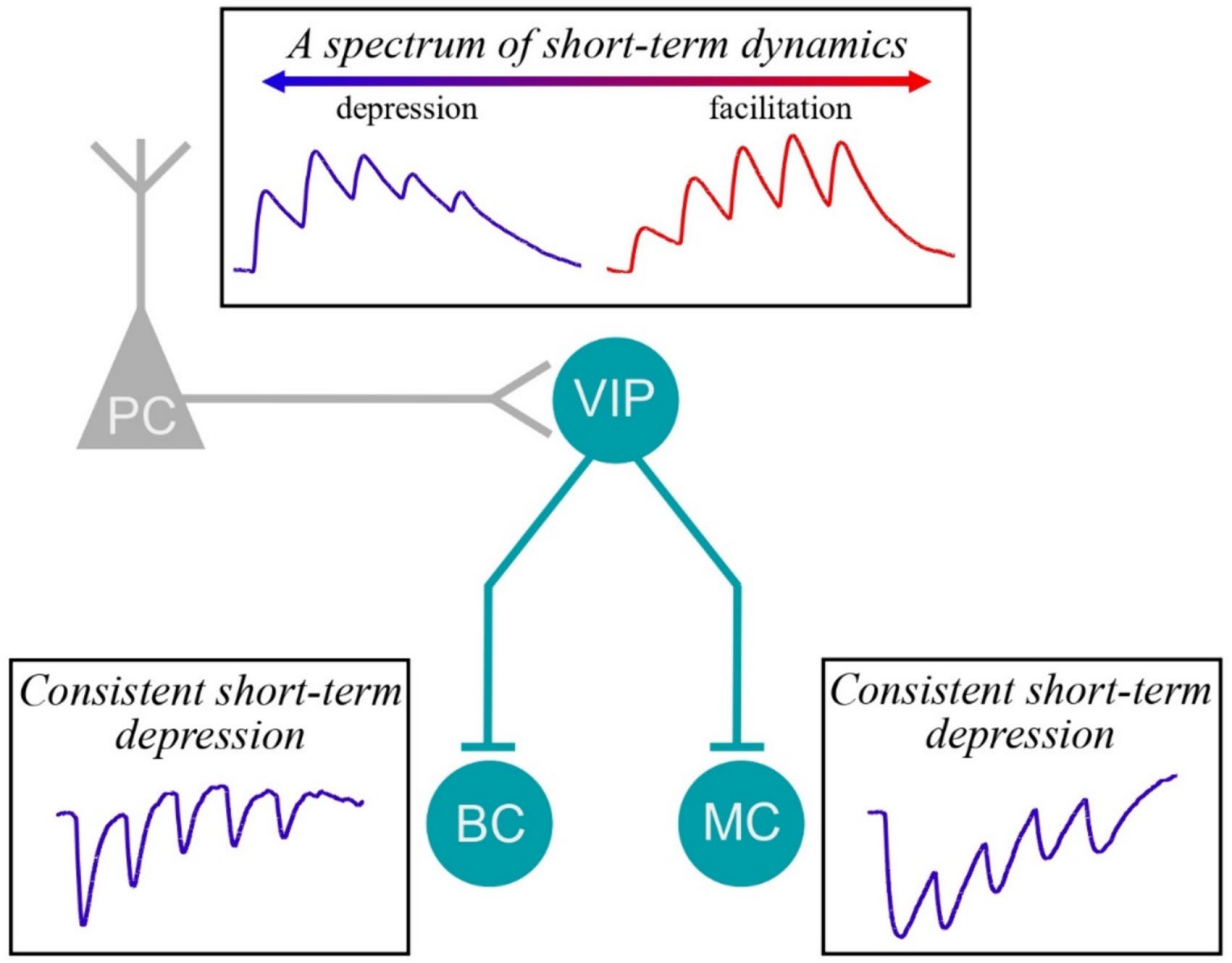
Short-term plasticity at VIP IN inputs and outputs. We found that E → VIP IN synapses were heterogeneous, showing instances of facilitation as well as of depression. However, VIP IN→MC and VIP IN→BC synapses were consistently short-term depressing.

Short-term plasticity is a synaptic feature that is important for information processing and E/I balance in the brain (Blackman et al., 2013). Here, we explored the short-term dynamics at VIP IN inputs and outputs at translaminar connections in the mouse motor cortex. We found that VIP IN outputs were consistently short-term depressing, whereas VIP IN inputs surprisingly displayed heterogenous short-term dynamics (Figure 5). Together, our findings highlight that short-term plasticity at VIP IN synapses is specific to synapse type and target cell (Blackman et al., 2013).

### Consequences of short-term plasticity

One of the key functional consequences of short-term plasticity is its role in modulating information flow across the synapse (Blackman et al., 2013). For example, whereas short-term depressing synapses are low-pass filters, short-term facilitating synapses are high-pass filters (Dittman et al., 2000). The impact of short-term plasticity will additionally depend on factors like cell type and synapse type. For example, short-term dynamics at E → E or E → I synapses will determine how quickly the postsynaptic cell will reach suprathreshold responses and spike, while short-term dynamics at I → E or I → I synapses will determine how quickly the postsynaptic cell will be inhibited.

In our study, we found that VIP IN→MC and VIP IN→BC synapses were short-term depressing, which suggests that these synapses would be rapidly shut down during periods of high-frequency activity. Thus, these synapses may be optimized for transferring information at low frequencies. Given that VIP IN→MC and VIP IN→BC synapses have a disinhibitory effect in cortical circuits, short-term depression may be helpful for maintaining E/I balance by ensuring that I → I → E disinhibition does not go awry. Disruptions in the short-term dynamics at VIP IN outputs may thus lead to states of hyperexcitability, e.g., seizures.

Though there are many factors that influence short-term plasticity outcomes, short-term plasticity is often stereotyped for a given synapse type (Blackman et al., 2013). We were therefore surprised to find a broad diversity in short-term dynamics at E → VIP IN synapses. VIP IN-mediated disinhibition plays an important role in promoting motor learning (Adler et al., 2019; Ren et al., 2022) as well as gating (Williams and Holtmaat, 2019) and enhancing (Fu et al., 2014; Fu et al., 2015) synaptic plasticity. VIP INs have also been shown to respond to reward in associative learning (Lee et al., 2022). Therefore, it may be advantageous for VIP INs to be tuned to respond to a wide spectrum of inputs. We propose that the broad diversity in short-term plasticity at E → VIP IN synapses could serve as a basis for encoding diverse signal dynamics relevant to behavioral learning.

### Disagreements surrounding the short-term dynamics VIP IN→MC synapses

In agreement with previous studies in cortex (Pi et al., 2013; Campagnola et al., 2022), we found that VIP IN→BC connections in the motor cortex exhibited short-term depression. At VIP IN→MC connections, however, there seems to be a discrepancy in short-term plasticity outcomes. In agreement with Pi et al. (2013), we found that VIP IN→MC synapses were short-term depressing. Other studies, however, have described VIP IN→MC synapses as short-term facilitating (Walker et al., 2016; Campagnola et al., 2022).

There are several candidate explanations for the apparent disagreement in these findings. Firstly, whereas we explored short-term plasticity at connections between L2/3 VIP INs and L5 MCs, studies that reported short-term facilitation were performed at VIP IN→MC synapses in L2/3 of the cortex (Walker et al., 2016; Campagnola et al., 2022). Given that short-term plasticity is dependent on cortical layer (Reyes and Sakmann, 1999; Chou et al., 2023), it is thus possible that short-term plasticity differs at L2/3 VIP IN connections to L2/3 MCs versus L5 MCs.

Secondly, the discrepancy in short-term plasticity outcomes at VIP IN→MC synapses could be explained by the different cortical regions in which these plasticity rules were tested. Short-term depression at VIP IN→MC synapses was described in the motor cortex (Figure 1F) and the auditory and medial prefrontal cortices (Pi et al., 2013), while short-term facilitation was described in the somatosensory cortex (Walker et al., 2016) and visual cortex (Campagnola et al., 2022). Since short-term plasticity outcomes have been shown to vary with brain region (Wang et al., 2006), it is perhaps not surprising if short-term plasticity at VIP IN→MC synapses differ across brain regions.

Lastly, differences in experimental methods that were used to study short-term dynamics at VIP IN→MC synapses may contribute to the discrepancy in short-term plasticity outcomes. For example, paired recordings were used in the studies that showed short-term facilitation at VIP IN→MC synapses (Walker et al., 2016; Campagnola et al., 2022), whereas studies that showed short-term depression at VIP IN→MC synapses relied on optogenetic activation of presynaptic VIP INs (Figure 1) and (Pi et al., 2013). ChR2 is known to flux calcium in addition to other cations (Yang et al., 2019) which could increase the probability of release and push short-term dynamics toward depression (Zhang and Oertner, 2007; Cruikshank et al., 2010; Jackman et al., 2014). However, we found that PPR measurements were indistinguishable when acquired by optogenetic versus extracellular stimulation (Figure 1F).

In conclusion, though we found that VIP IN outputs exhibited stereotypic short-term depression, others have reported instances of short-term facilitation at, e.g., VIP IN→MC synapses. Further studies are required to clarify this apparent discrepancy in short-term plasticity dynamics at VIP IN outputs across studies.

### Diverse E → VIP IN short-term dynamics associated with target cell

We found that some VIP INs received robust facilitating inputs while others received a mix of facilitating and depressing inputs, suggesting that the heterogeneous short-term dynamics at VIP IN inputs were associated with the target VIP IN cell rather than with the presynaptic inputs. This outcome is consistent with the existence of two different VIP IN types that receive either facilitating or depressing inputs. However, we also found that short-term facilitating versus short-term depressing VIP INs were indistinguishable with respect to morphology (Supplementary Figure S2) and electrophysiology (Supplementary Table S1), indicating that the different dynamics cannot simply be attributed to different VIP IN subtypes. For example, perhaps activity-dependent learning rules promote facilitation or depression in different VIP IN cells, yet they are all of the same type.

How might postsynaptic VIP INs influence short-term dynamics? One possible explanation could be that a subset of VIP INs co-express other molecules that influence synaptic dynamics. Indeed, several studies have shown that cholinergic cells are also VIP-positive (Eckenstein and Baughman, 1984; von Engelhardt et al., 2007; Granger et al., 2020). Cholinergic cells have also been shown to be mainly located in L2/3 of the cortex (von Engelhardt et al., 2007), which is characteristic of VIP IN populations (Prönneke et al., 2015; McFarlan et al., 2024). Cholinergic VIP cells have been shown to provide direct excitation rather than disinhibition, suggesting that they may serve a different role (Obermayer et al., 2019). Furthermore, in agreement with our findings at E → VIP IN synapses, excitatory connections onto cholinergic cells have also been shown to exhibit both short-term facilitation and short-term depression (von Engelhardt et al., 2007). Thus, the heterogeneous short-term dynamics at VIP IN inputs may be explained by the co-expression of acetylcholine in a subset of VIP INs. In this view, there are two different VIP IN types, cholinergic and non-cholinergic, but they are not readily distinguishable by morphometry or electrophysiology.

Another possible explanation for the diversity in short-term dynamics at E → VIP IN synapses may be the expression of postsynaptic proteins that regulate presynaptic release probability. At E → I connections in the hippocampus, for example, short-term plasticity outcomes were shown to be regulated by postsynaptic expression of proteins that controlled the presynaptic probability of release (Sylwestrak and Ghosh, 2012). Whereas excitatory connections onto parvalbumin-expressing INs were short-term depressing with a high probability of release, E → oriens-lacunosum molecular (OLM) IN synapses were short-term facilitating with a low probability of release. The postsynaptic expression of the Extracellular Leucine-rich repeat Fibronectin containing 1 (Elfn1) protein from OLM INs regulated the probability of release in presynaptic excitatory cells (Sylwestrak and Ghosh, 2012). Given that computational modeling in our study revealed a wide variability in probability of release at E → VIP IN synapses and short-term plasticity diversity was associated with postsynaptic cell type, rather than input type, it is possible that postsynaptic VIP INs may be regulating the probability of release at presynaptic excitatory inputs. We note that several of the above interpretations are not mutually exclusive.

### Caveats

One caveat of our study is the use of extracellular stimulation to explore short-term plasticity at VIP IN→MC and VIP IN→BC synapses. Though excitatory synaptic transmission was blocked during these experiments, we could not be sure that we did not activate other L2/3 INs in addition to VIP INs. Indeed, there are many different L2/3 IN subtypes, some of which have descending axons like VIP INs that may reach L5 (Gouwens et al., 2020). However, having a descending axon does not necessarily mean these INs innervate L5 MCs and BCs.

Moreover, the use of extracellular stimulation in L2/3 likely recruited multiple excitatory neurons, which like INs may also be heterogenous (Tasic et al., 2018; Gouwens et al., 2019). Such a heterogeneity of PC subtypes could contribute to the diverse E → VIP IN short-term dynamics we observed.

The use of optogenetic activation of ChR2 to study short-term plasticity at VIP IN outputs is another possible caveat in our study. Since ChR2 is known to flux calcium (Nagel et al., 2003), it is possible that ChR2 activation may increase calcium influx at the synaptic terminal and thus increasing the probability of release and biasing the synapse toward short-term depression. However, this seemed unlikely given that presynaptic laser stimulation occurred in L2/3, hundreds of micrometers away from postsynaptic cells in L5. In agreement, we found no difference in short-term dynamics at VIP IN outputs when activating presynaptic L2/3 VIP INs optogenetically or using extracellular stimulation (Figure 1F).

The use of artificial solutions in the acute slice is a final potential caveat. Extracellular calcium concentration used in ACSF is typically higher than what is observed in physiological conditions (Ding et al., 2016). Using a higher concentration of extracellular calcium is commonly used for studies of plasticity (Lu et al., 2007; Sarihi et al., 2008; Sarihi et al., 2012; Pi et al., 2013; Walker et al., 2016), since it is known to enhance synaptic transmission (Wang and Lu, 2023). However, higher extracellular calcium may also alter intrinsic neuronal properties (Wang and Lu, 2023) and short-term plasticity (Borst, 2010). Using physiological calcium concentrations *in vitro* yields short-term plasticity data more closely resembling those found *in vivo* (Borst, 2010). Thus, using physiological calcium concentrations *in vitro* may be important for comparisons between *in-vitro* and *in-vivo* studies.

### Outlook and future directions

In summary, our study suggests that short-term dynamics at VIP IN inputs and outputs are specific to synapse type. Although we covered new ground by characterizing otherwise poorly studied synapse types, this finding was in and of itself not surprising, as both long and short-term synaptic plasticity are often specific to synapse type (Blackman et al., 2013; McFarlan et al., 2023). It remains unclear, however, why there was a broad diversity in short-term plasticity at E → VIP IN synapses, although we were able to link diversity to the postsynaptic cell.

One interpretation is that there are two or more classes of motor cortex L2/3 VIP INs (He et al., 2016; Tasic et al., 2018; Gouwens et al., 2020), but another not necessarily mutually exclusive explanation is that the functionality of VIP INs requires that their inputs cover a full spectrum of synaptic dynamics. This scenario would be a short-term plasticity equivalent to that described for long-term plasticity at parallel fiber inputs to cerebellar Purkinje cells, where the learning rules are heterogeneous to accommodate a diversity of behavioral outputs (Suvrathan, 2019). In this view, the broad diversity in short-term plasticity of VIP IN inputs would form a basis to code for a broad range of contrasting signal dynamics. Future work is necessary to clarify this curious diversity of E → VIP IN synapses.

## Data Availability

The raw data supporting the conclusions of this article will be made available by the authors, without undue reservation.
