# Supplementary material for: The short-term plasticity of VIP interneurons in motor cortex
Source: Front Synaptic Neurosci. 2024 Aug 29;16:1433977. doi: 10.3389/fnsyn.2024.1433977 (PMC11390561; doi:10.3389/fnsyn.2024.1433977)
Supplement: Supplementary file 1 [file Data_Sheet_1.PDF]

Supplementary Material

The Short-Term Plasticity of VIP Interneurons in Motor Cortex

Amanda R. McFarlan[1,2], Isabella Gomez[1], Christina Y.C. Chou[1,2], Adam Alcolado[3], Rui Ponte Costa[4], P. Jesper Sjöström[1*]

[1] Centre for Research in Neuroscience, Brain Repair and Integrative Neuroscience Program, Department of Neurology and Neurosurgery, The Research Institute of the McGill University Health Centre, Montreal General Hospital, Montreal, QC, Canada

[2] Integrated Program in Neuroscience, McGill University, Montreal, QC, Canada

[3] MTL.AI Inc, Montreal, QC, Canada

[4] Centre for Neural Circuits and Behaviour, Department of Physiology, Anatomy and Genetics, Medical Sciences Division, University of Oxford, Oxford, United Kingdom

*** Correspondence:** jesper.sjostrom@mcgill.ca

1 Supplementary Figures

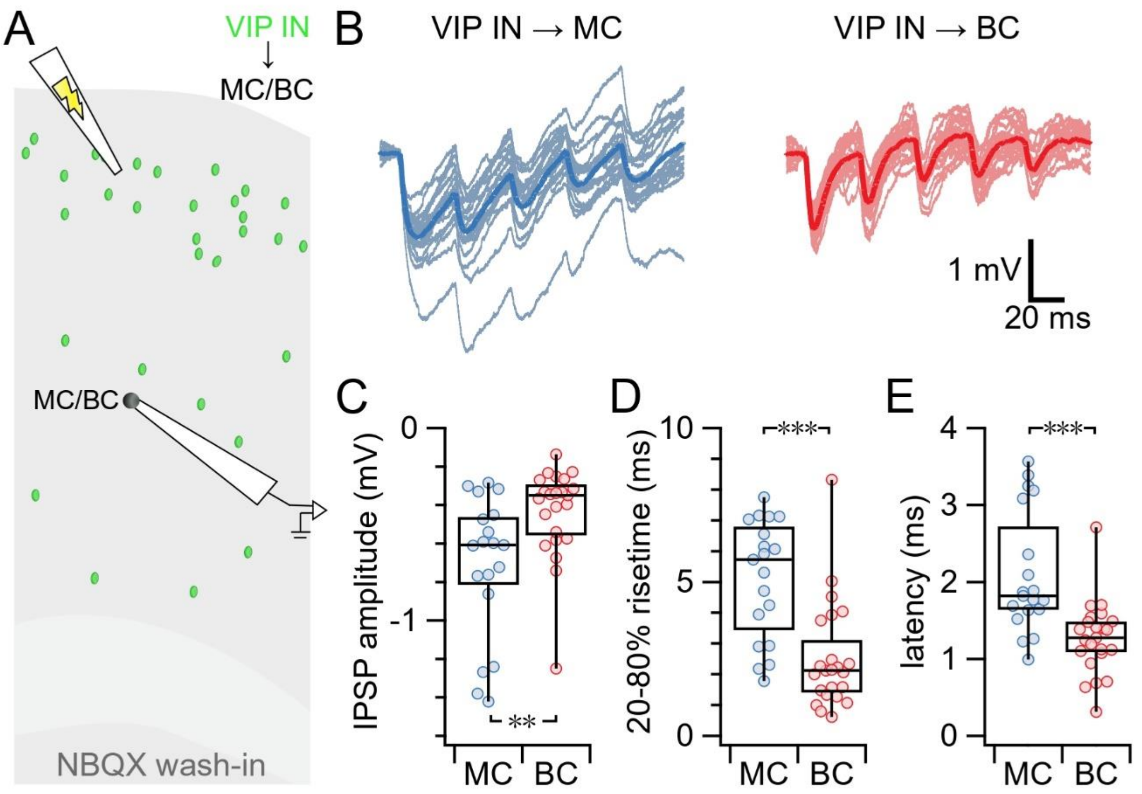

Supplementary Figure 1. VIP IN outputs exhibited differing kinetics. (A) Schematic illustrating the experimental paradigm. MCs and BCs were targeted for whole-cell recording in L5 of the mouse motor cortex. L2/3 VIP INs were activated with extracellular stimulation which resulted in IPSPs in connected L5 MCs and BCs. Bath application of NBQX was used to block excitatory transmission. **(B)** Sample traces illustrating recorded IPSPs in a patched MC and BC following 5 pulses of extracellular stimulation delivered at 30 Hz. Average traces are in blue (MC) and red (BC) while individual responses are in light blue (MC) and pink (BC). **(C)** VIP IN→MC (n = 19 connections, N = 3 animals) and VIP IN→BC synapses (n = 23 connections, N = 3 animals) differed in kinetics. IPSP amplitude was larger for VIP IN→MC synapses compared to VIP IN→BC synapses (VIP IN→MC: -0.71 mV ± 0.1 mV vs. VIP IN→BC: -0.44 mV ± 0.05 mV, t-test $p < 0.01$). Compared to VIP IN→MC synapses, VIP IN→BC synapses had a shorter **(D)** 20-80% rise time (VIP IN→MC: 5.1 ms ± 0.4 ms vs. VIP IN→BC: 2.5 ms ± 0.4 ms, t-test $p < 0.001$) and **(E)** latency (VIP IN→MC: 2.1 ms ± 0.2 ms vs. VIP IN→BC: 1.3 ms ± 0.1 ms, unequal variances t-test $p < 0.001$). Rise time, latency, and amplitude were analyzed based on averages from individual connections.

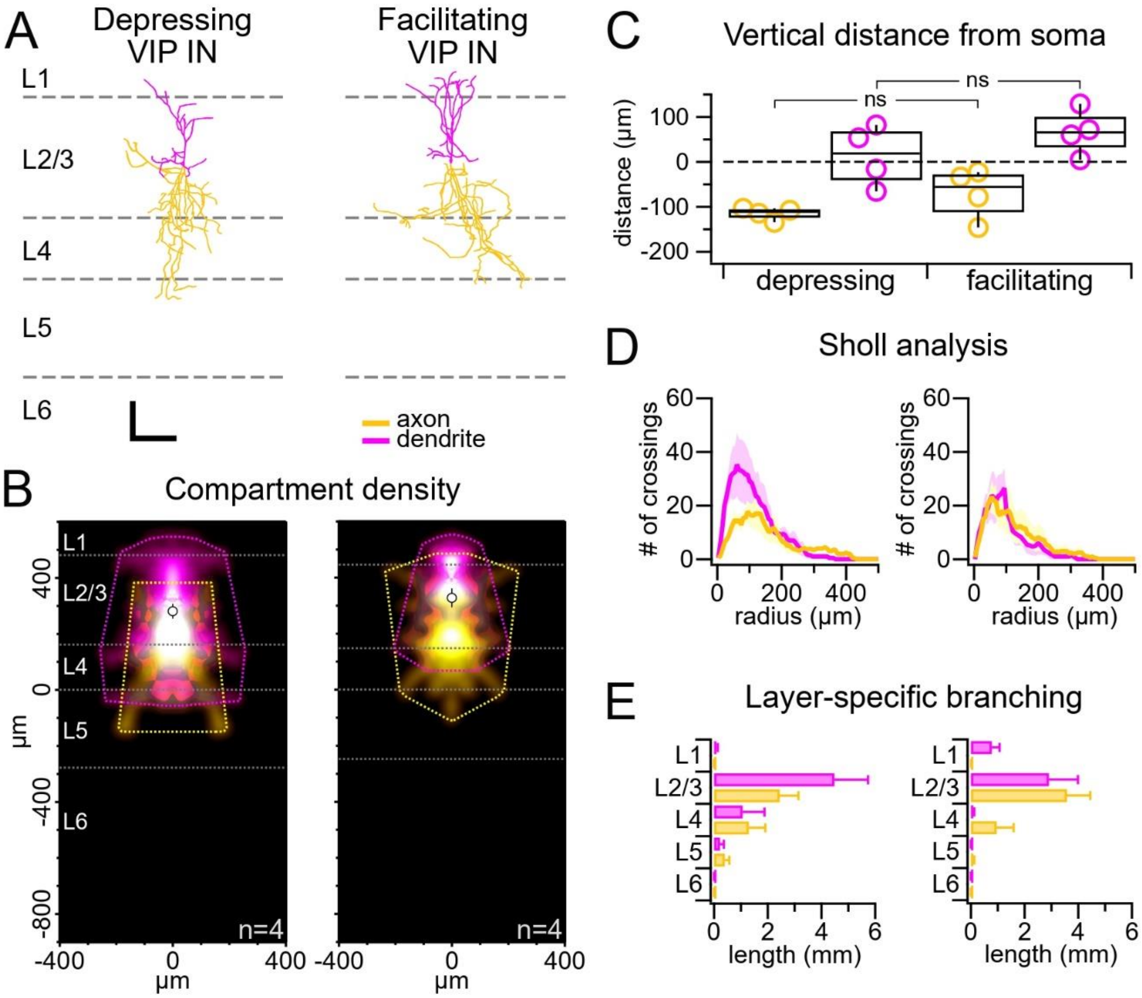

Supplementary Figure 2. VIP INs with depressing vs. facilitating inputs were morphologically indistinguishable. (A) Sample reconstructions from a short-term depressing L2/3 VIP IN (left) and from a short-term facilitating L2/3 VIP IN (right). E→VIP IN synapses with a short-term depression (STD) index < 1 were categorized as short-term depressing, whereas E→VIP IN synapses with an STD index > 1 were categorized as short-term facilitating. The STD index was calculated as the average of $EPSP_{3+4+5}$ divided by $EPSP_1$. Axons are labeled yellow and dendrites are labeled pink. Scale bar is 100 µm for both axes. **(B)** Compartment density heat map for short-term depressing VIP INs (left: n = 4 cells, N = 4 animals) and short-term facilitating VIP INs (right: n = 4 cells, N = 4 animals). **(C)** Comparing the axonal and dendritic compartment center of mass vertically, we found no difference between short-term depressing and facilitating VIP INs in axonal branching (depressing: -115 µm ± 7 µm vs. facilitating: -70.2 µm ± 30 µm, unequal variances t-test p = 0.21) or dendritic branching (depressing: 13 µm ± 30 µm vs. facilitating: 66 µm ± 30 µm, t-test p = 0.26). **(D)** Sholl analysis and **(E)** layer-specific branching highlighted how facilitating and depressing VIP INs have similar morphologies.

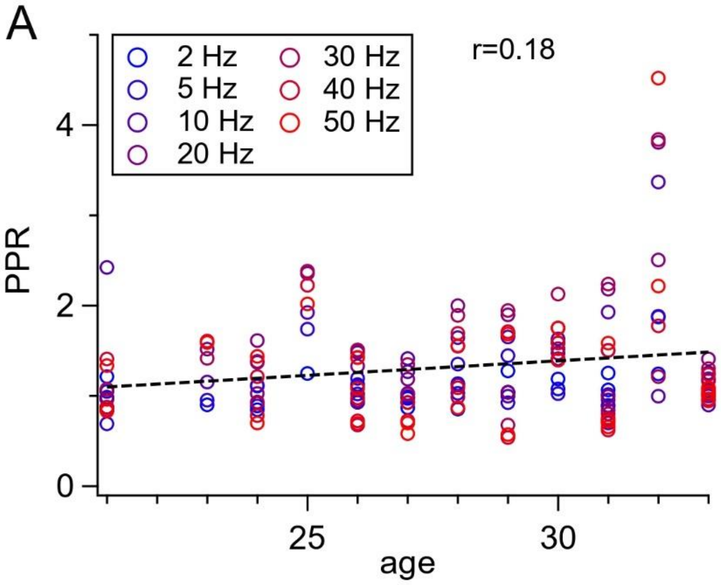

Supplementary Figure 3. PPR at VIP IN inputs did not correlate with age. (A) We used a linear mixed model to test the dependency of PPR on age for all combined frequencies (2, 5, 10, 20, 30, 40, and 50 Hz; n = 25 cells, N = 18 animals). We found that PPR did not depend on age (p = 0.42).

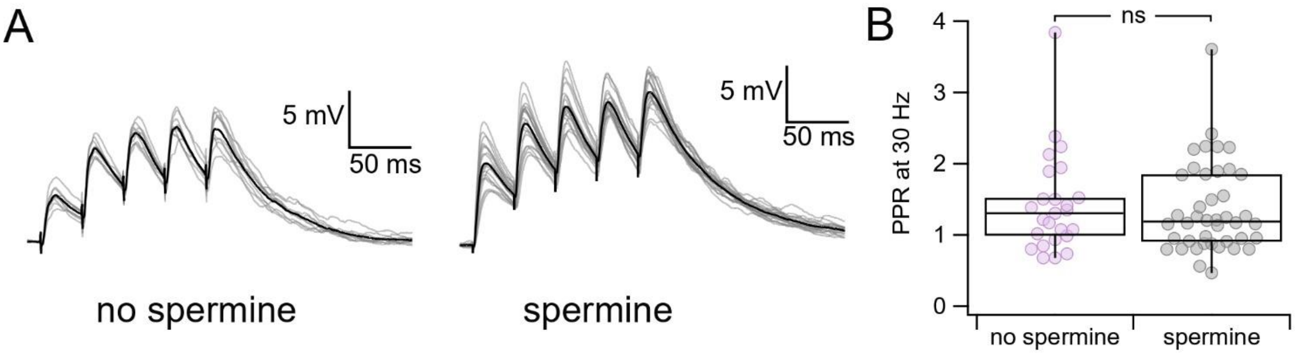

Supplementary Figure 4. VIP INs did not signal via calcium-permeable AMPA receptors. (A) Sample traces illustrating EPSPs in response to extracellular stimulation in a patched VIP IN with regular internal solution (left: labeled “no spermine”) and a patched VIP IN supplemented with spermine in the internal solution (right: labeled “spermine”). Gray traces represent individual responses and black trace represents the average response. **(B)** Linear mixed model statistics revealed that PPR at 30 Hz did not differ between the no spermine and spermine conditions (PPR at 30 Hz no spermine: 1.4 ± 0.1, n = 25 connections, N = 18 animals, vs. spermine: 1.4 ± 0.1, n = 42 connections, N = 6 animals; p = 0.67).

2 Supplementary Tables

Supplementary Table 1. Electrophysiological properties of depressing vs. facilitating L2/3 VIP INs in the motor cortex were indistinguishable. E→VIP IN synapses with a short-term depression (STD) index < 1 were categorized as short-term depressing, while E→VIP IN synapses with an STD index > 1 were categorized as short-term facilitating. STD index was calculated as the average of $EPSP_{3+4+5}$ divided by $EPSP_1$. All p-values were obtained with Student's t-test, except for rheobase, for which Wilcoxon-Mann-Whitney two-sample rank test was used.

**Property**	**Short-term depressing**	**Short-term facilitating**	**p-value**
Resting potential (mV)	-60 ± 1	-61 ± 3	0.83
Spike threshold (mV)	-36 ± 1	-38 ± 2	0.37
Spike height (mV)	28 ± 3	30 ± 3	0.69
Spike half width (ms)	1.1 ± 0.1	1.2 ± 0.2	0.46
Rheobase (pA)	81 ± 10	150 ± 30	0.051
Membrane time constant (ms)	19 ± 1	17 ± 2	0.30
Input resistance (MΩ)	280 ± 20	220 ± 30	0.13
STD index at 30 Hz	0.55 ± 0.1	1.5 ± 0.1	<0.001
Spike patterns:<br>Adapting<br>Bursting<br>Irregular	 9 (69%)<br>3 (23%)<br>1 (8%)	 8 (67%)<br>1 (8%)<br>3 (25%)	0.53
n cells	13	12	-
